# Profitability and Market Value of Orphan Drug Companies: A Retrospective, Propensity-Matched Case-Control Study

**DOI:** 10.1371/journal.pone.0164681

**Published:** 2016-10-21

**Authors:** Dyfrig A. Hughes, Jannine Poletti-Hughes

**Affiliations:** 1 Centre for Health Economics and Medicines Evaluation, Bangor University, Ardudwy, Holyhead Road, Bangor, LL57 2PZ, United Kingdom; 2 University of Liverpool Management School, University of Liverpool, Chatham Street, Liverpool, L69 7ZH, United Kingdom; H Lee Moffitt Cancer Center and Research Institute, UNITED STATES

## Abstract

**Background:**

Concerns about the high cost of orphan drugs has led to questions being asked about the generosity of the incentives for development, and associated company profits.

**Methods:**

We conducted a retrospective, propensity score matched study of publicly-listed orphan companies. Cases were defined as holders of orphan drug market authorisation in Europe or the USA between 2000–12. Control companies were selected based on their propensity for being orphan drug market authorisation holders. We applied system General Method of Moments to test whether companies with orphan drug market authorization are valued higher, as measured by the Tobin’s Q and market to book value ratios, and are more profitable based on return on assets, than non-orphan drug companies.

**Results:**

86 companies with orphan drug approvals in European (4), USA (61) or both (21) markets were matched with 258 controls. Following adjustment, orphan drug market authorization holders have a 9.6% (95% confidence interval, 0.6% to 18.7%) higher return on assets than non-orphan drug companies; Tobin’s Q was higher by 9.9% (1.0% to 19.7%); market to book value by 15.7% (3.1% to 30.0%) and operating profit by 516% (CI 19.8% to 1011%). For each additional orphan drug sold, return on assets increased by 11.1% (0.6% to 21.3%), Tobin’s Q by 2.7% (0.2% to 5.2%), and market to book value ratio by 5.8% (0.7% to 10.9%).

**Conclusions:**

Publicly listed pharmaceutical companies that are orphan drug market authorization holders are associated with higher market value and greater profits than companies not producing treatments for rare diseases.

## Introduction

The Orphan medicinal products regulation of the European Parliament and the United States Orphan Drug Act aim to incentivise pharmaceutical companies to develop medicines for rare diseases that would otherwise not be commercially viable [1]. A measure of the success of these regulations is the number of orphan drugs approved. The European Commission has designated 1,586 products as orphan, and authorized 123 since legislation was introduced in 2000. The Food and Drug Administration (FDA) in the USA has approved 503 drugs and biologic products for rare diseases since the 1983 Orphan Drug Act, compared with fewer than 10 such products in the decade prior to the Act [2]. Orphan drugs accounted for nearly half (21 of 45) of all new innovative drugs approved by the FDA in 2015 [3].

To qualify for orphan designation in the European Union, a medicine must be indicated for a life-threatening or chronically debilitating disease with either a prevalence of less than 5 in 10,000 or otherwise deemed to be not profitable to develop. There must be no satisfactory alternative treatment, or the medicine must be of significant benefit to those affected by the condition. The FDA Office of Orphan Products Development may designate a drug as an orphan if indicated for a condition with a prevalence of <200,000 people in the USA.

The small markets associated with rare diseases, however, necessitate high prices for returns to be made on investments, and orphan drugs are generally more expensive than non-orphan drugs [4]. Each one of the world’s 10 most expensive drugs is an orphan, with alipogene tiparvovec (gene therapy approved in Europe for inherited lipoprotein lipase deficiency) ranked highest at about US$1.4m per patient over a year [5]. The revenue-generating potential of orphan drugs is consequently as great as for non-orphan drugs [6] with almost a third being US$1bn blockbuster products in terms of global annual sales [7]. The orphan drugs market is expected to reach US$176bn by 2020, and account for 19% of total branded prescription drug sales [4].

Orphan drugs also command a higher profit margin, owing to shorter clinical development time, incentives related to research and development, reduced marketing costs and premium pricing [6,8]. However, it is unclear whether this necessarily translates to higher company profits [9,10]. One cross-sectional analysis of a small sample of specialised orphan drug companies concluded that they had not performed as strongly as other companies [11]. This observation is not supported by the evidence of the rapid and extensive diversification into the orphan drugs market by large pharmaceutical companies, some of which have established dedicated rare disease units, and acquired or partnered biotech companies already in the rare disease sector [12].

Concerns have been expressed meanwhile that orphan drug policies are being exploited by companies as treatments initially approved for rare diseases are later used more broadly [13]. Rituximab, as one example, was initially approved as an orphan drug by the FDA for the treatment of follicular non-Hodgkin’s lymphoma. It is now used to treat a wide variety of conditions making it the fourth best-selling drug in the world in 2014.

We hypothesise that companies with orphan drug market authorization are more profitable and are more attractive investment opportunities than non-orphan drug companies. We aimed to test whether the financial performance of publicly listed European and US orphan drug companies is better than matched non-orphan drug companies in terms of their market value and profitability.

## Methods

### Source data and sample

Cases were defined as publicly-listed companies which were holders of orphan drug market authorisation in or Europe or the USA in any period between 2000 (when the Orphan Medicinal Product Regulation was introduced in Europe) and 2012. These were identified from the European Medicines Agency database of authorised orphan medicines [14] and from the US Food and Drug Administration’s database of Orphan Drug Product designation [15].

We defined potentially eligible controls as publicly-listed companies, registered in the same countries as the cases, but which had no market authorisation for orphan medicinal products in Europe or the USA during the sampling period. These were identified from all publicly-trading listed companies within the pharmaceutical and biotechnology industrial subsectors (Datastream, Thomson Reuters, NY), and cross-checked with the orphan drugs databases. Companies were required to have complete data on the relevant financial variables.

Control companies were matched based on their propensity for being orphan drug market authorisation holders, conditional on a set of variables using the *psmatch2* command in Stata version 13 (Statacorp, TX) [16]. Propensity scores were calculated using a Probit model with robust standard errors adjusted with company clusters using the following explanatory variables (Table 1): the natural logarithm of total assets, the ratio of research and development expenditure to total assets, and a dummy to identify a company as being in the pharmaceutical subsector. We selected the best matching companies, based on the largest year-averaged propensity scores by country, to achieve a 3:1 ratio of control to cases. A sample based on matching by maximum propensity score in any given year by country, was assessed as an alternative approach, as was consideration of total revenue (expressed as a ratio to total assets).

**Table 1:** Definition of the variables.

Variables	Description
*Dependent variables*	
Ln Tobin’s Q ratio ln(TQ)	A measure of a company’s market value, calculated as the natural logarithm of the ratio of total assets minus book value of companies’ equity plus market value of equity, to total assets.
Ln Market to book value ratio ln(MB)	A measure of a company’s market value, calculated as the natural logarithm of the ratio of market value of a company’s equity to the book (or accounting) value of equity.
Return on Assets (ROA)	A profitability ratio which gauges a company’s return on investment. Calculated as the ratio of a company’s net income prior to financing costs (earnings before interest and taxes) to total assets.
Operating profit (EBITDA/REV)	An alternative profitability ratio which gauges a company’s return on investment. Calculated as the ratio of a company’s net income prior to financing costs (earnings before interest, tax, depreciation and amortization) to total revenue.
*Key variables*	
ORPHAN	Dummy variable that equals one for the year in which a company holds market authorization for an orphan drug, otherwise zero.
NORPHAN	Number of orphan drugs with authorization in a given year.
DORPHAN	Dummy variable that equals one if a company has held orphan drug market authorization at any time, otherwise zero.
SORPHAN	Ratio of orphan drug sales to total sales.
*Financial Variables*	
Size	Size is likely to impact on performance as a result of scale differences in operations, market regulations, and agency problems. Calculated as the natural logarithm of total assets, converted from local currencies to US dollars using historic foreign exchange rates.
Leverage	Debt can play a role in reducing the agency costs of free cash flows by preventing investments in non-positive net present value projects. By contrast, debt might also increase the likelihood of bankruptcy and credit risks, which may prevent a company from investing in profitable investment opportunities. Calculated as the ratio of total debt to total assets.
R&D/TA	Research and development activities result in new technologies, products or production processes that would return gains in performance. Represented as the ratio of research and development expenditure to total assets.
Capex/PPE	The ratio of annual capital expenditure to the value of existing property, plant and equipment, represents a company’s investment intensity.

### Outcomes and possible confounding factors

We assessed the performance of companies based on their Tobin’s Q ratio, market to book value ratio and return on assets (Table 1). The primary explanatory variable of interest (ORPHAN) is a time-variant dummy that equals one for a given year in which a company holds orphan drug market authorization, otherwise zero.

Potential confounding variables included company size, leverage, ratio of research and development expenditure to total assets, and the ratio of capital expenditure to property, plant and equipment (Table 1).

Financial data for all companies were obtained from annual accounts for the years 2000–12 from Datastream. These were each Winsorized at 98% to limit the influence of outliers.

### Data analysis

We used a standard model of company performance based on a linear function of financial explanatory variables (Table 1) [17–20]. Corporate performance models are likely to present problems of endogeneity arising from three sources [20]: i) simultaneity of dependent and explanatory variables e.g. performance drives size and *vice versa*; ii) the correlation of regressors and error terms, e.g. shocks affecting corporate performance are also likely to affect other explanatory variables such as leverage; and, iii) the likely dynamic relationship between current explanatory variables with past performance. To overcome these problems, we applied system General Method of Moments (GMM) using the Stata command *xtabond2* to obtain the estimates from the dynamic specification of the performance model (i.e. including the lagged dependent variable) [21]. We specified the function for *small* sample adjustment, the *twostep* command to correct for finite-sample bias and we used robust standard errors which are consistent with panel-specific autocorrelation and heteroscedasticity in the one-step estimation. We treated the value model conservatively by assuming that all the financial variables were endogenous, leaving the time dummies and ORPHAN as exogenous variables. We instrumented the endogenous variables by using as many lags as possible to avoid weakening the Hansen test of the instruments’ joint validity. Not all the available instruments were used to specify the regressions (i.e. from lags two, onwards) as this would weaken the Hansen test of overriding restrictions, and the difference-in-Hansen tests of exogeneity of instrument subsets for the equations in levels. We measured the presence of autocorrelation of second order in the disturbance term (AR2), which might indicate bias in the coefficients. The validity of the system GMM models was confirmed in all regressions.

### Sensitivity analyses

To test the robustness of the findings to modelling assumptions, we conducted several sensitivity analyses.

The pooled OLS estimator was used for comparison with the system GMM estimator, as while the latter may be more appealing statistically, it is very sensitive to the specification of the instrumental variables [22]. The OLS regressions were calculated with robust standard errors clustered by company and included country and time dummies to account for economic factors that were outside the control of companies.

The variable ORPHAN was specified in alternative ways; firstly as a discrete variable to represent the annual number of orphan drugs sold by each company (NORPHAN) to test whether an increase in the number of orphan drugs per company is associated with better performance. Secondly, we applied the Taylor and Hausman estimator which controls for fixed effects without eliminating the time-invariant effects, by specifying the DORPHAN variable, which equals one if the company holds market authorization for an orphan drug at any time in the sampling period, otherwise zero. Thirdly, we tested whether increases in orphan drug sales corresponded with increases in company performance by constructing a variable to represent orphan drug sales as a proportion of each company’s total annual sales (SORPHAN) for the period 2004–12. Orphan drug sales were obtained from the DrugAnalyst Database [23], and total sales from Datastream for each corresponding year. As orphan drug sales were not available for all companies, we used a different set of control companies based on propensity score matching, as before.

## Results

We identified 181 companies with authorisation for orphan medicinal products in US (135), European (20) or both (26) markets. Of these, 93 companies were excluded: 80 were not publicly listed, 9 were registered in jurisdictions outside of Europe or the USA and 6 did not have the relevant financial data available. The remaining 86 companies, with orphan drug approvals in the US (61), European (4) or both (21) markets, were registered in 7 countries: Denmark, France, Germany, Spain, Switzerland, United States and the UK (Table 2). These companies were market authorization holders for an average of 2 (range 1 to 13) orphan drugs and for 197 (63%) of the 313 orphan drugs authorized in total over the sampling period. Orphan drug sales represented 38% of total sales in the 28 companies for which we had data, but this ranged from a mean of 6% among French companies to 53% in Swiss companies. Within individual companies, orphan drug sales ranged from 0.1% to 100% of total sales.

**Table 2:** Characteristics of the data.

Country	Number of non-orphan companies (unmatched sample)	Number of orphan drug companies	Mean number (SD; range) of orphan drugs per company (in any single year)	Orphan drug sales as a proportion of total sales, reported as mean (SD; range)*
Denmark	14	3	2.24 (0.83; 1, 3)	0.20 (0.07; 0.017, 0.23)
France	30	4	1.75 (0.53; 1, 3)	0.06 (0.08; 0.003, 0.20)
Germany	32	3	1.79 (0.92; 1, 3)	0.18 (0.07; 0.115, 0.28)
Spain	6	1	1.83 (0.41; 1, 2)	n/a
Switzerland	19	3	4.00 (1.58; 1, 8)	0.53 (0.45; 0.047, 0.98)
UK	107	5	3.24 (2.25; 1, 8)	0.20 (0.19; 0.001, 0.52)
USA	683	67	1.70 (1.30; 1, 9)	0.44 (0.38; 0.001, 1.00)
All countries	891	86	2.02 (1.51; 1, 9)	0.38 (0.37; 0.001, 1.00)

* Data available for 28 companies; no orphan drug sales data available for the Spanish company.

The characteristics of case and control companies are presented in Table 3. Matching resulted in a less biased selection of controls, as indicated by the standardised percentage bias (which is the difference of the means between case and control samples, expressed as a percentage of the square root of the average of the sample variances in each group [24]). Although matching based on the maximum propensity score achieved an agreement rate of 71.7% with companies selected on the basis of average score, the sample was more biased and therefore not considered suitable for further analysis (S1 Table). Inclusion of the ratio of total revenue to total assets in calculating propensity score resulted in a selection of controls that only differed by one company (agreement rate of 99.6%).

**Table 3:** Mean values of financial variables for orphan and non-orphan drug companies.

	Orphan companies	Unmatched sample of non-orphan companies			Non-orphan companies matched by average propensity score		
Variables	Mean	Mean	P value	Bias (%)	Mean	P value	Bias (%)
*Performance measures*							
TQ	3.73	11.33	<0.001		5.08	0.204	
MB	4.94	3.43	0.090		4.37	0.363	
ROA	-0.15	-1.14	<0.001		-0.37	0.011	
EDITDA/REV	-7.65	-22.92	<0.001		-11.04	0.221	
*Explanatory variables*							
Size	12.02	10.19	<0.001	63.6	11.45	<0.001	20.3
Leverage	0.32	0.55	0.003	-14.5	0.31	0.772	1.3
R&D/TA	0.20	0.39	<0.001	-37.4	0.16	0.007	11.0
Capex/PPE	0.33	0.34	0.389	-3.6	0.31	0.324	4.4
Pharmaceutical sub-sector	0.57	0.44	<0.001	26.3	0.70	<0.001	-26.0
Mean bias (%)				29.1			12.6
Median bias (%)				26.3			11.0

P values indicate the significance of the difference in means between controls and the sample of orphan drug companies.

On average, orphan companies are larger in size and have a higher ratio of R&D expenditure to total assets than matched controls. A lower proportion of orphan companies are from the pharmaceutical subsector as opposed to the biotechnology subsector. Leverage and Capex/PPE were comparable between orphan and non-orphan companies. Orphan drug companies have comparable unadjusted TQs (3.73 vs 5.08) and MB (4.94 vs 4.37) to matched controls, but higher ROA (-0.15 vs -0.37) and EBITDA/REV (-7.65 vs -22.92) (Table 3).

Following adjustment for confounders, ORPHAN is positively significant in determining company performance (Table 4). Compared with non-orphan companies, the Tobin’s Q ratio of companies holding market authorisation for orphan drugs is higher by 9.9% (95% confidence interval, 1.0% to 19.7%); the market to book value ratio is higher by 15.7% (95% CI, 3.1% to 30.0%); ROA is higher by 9.6% (95% CI, 0.6% to 18.7%); and operating profit higher by 515.5% (CI, 19.8% to 1011%) (S2 Table).

**Table 4:** The impact of orphan drug company status on company performance.

	ln(TQ)		ln(MB)		ROA	
Variables	Estimator	P value	Estimator	P value	Estimator	P value
ORPHAN	0.094	0.026	0.146	0.013	0.118	0.038
Size	-0.008	0.479	0.018	0.297	0.076	0.148
Leverage	0.149	<0.001	1.069	0.001	-0.545	0.045
R&D/TA	0.246	0.024	0.380	0.062	-0.339	0.512
Capex/PPE	0.076	0.283	0.077	0.525	0.516	0.040
Dependent_t-1_	0.504	<0.001	0.535	<0.001	0.259	0.052
Constant	0.390	0.006	0.398	0.056	-1.227	0.144
Number of observations	2,390		2,141		2,411	
Number of cases: controls	84: 244		83: 229		83: 250	
Number of instruments	293		293		263	
AR(2) test		0.467		0.970		0.748
Hansen test		0.293		0.433		0.090
Difference-in-Hansen test		0.887		0.765		0.959

All regressions include time dummies. Instruments are set from t-2 to t-6 in the ln(TQ) and ln(MB) models and from t-3 to t-7 in the ROA model.

The number of companies included in the analyses was lower than the original sample of 86 cases and 258 controls. This was for two reasons: (i) system GMM requires at least two years of data, which were not available for some companies; and (ii) ln(MB) was undefined for companies reporting negative equity.

The AR(2) test reports the P value of a test for second-order serial correlation in the first-differenced residuals, under the null of no serial correlation. The Hansen test reports the P value of the test of over-identification under the null that all instruments are valid. The difference-in-Hansen test reports the P value of the null hypothesis that instruments used for the equations in levels are exogenous.

Capex/PPE is associated with increases in profitability, but neither Capex/PPE nor company size are significantly associated with market value. R&D/TA is positive and significant in determining the value of companies (i.e. ln(TQ) and ln(MB)), but not significant in determining ROA. An increase in leverage is associated with higher company value, but lower profitability.

### Sensitivity analyses

Use of pooled OLS supported the findings of system GMM with respect to the coefficient on the ORPHAN variable. However, some differences were apparent for other variables. OLS revealed significant associations between R&D/TA and ln(MB) and between Capex/PPE and market value (S3 Table). Associations between leverage and ln(MB) and between Capex/PPE and ROA were no longer significant in the pooled OLS.

**Fig 1:**
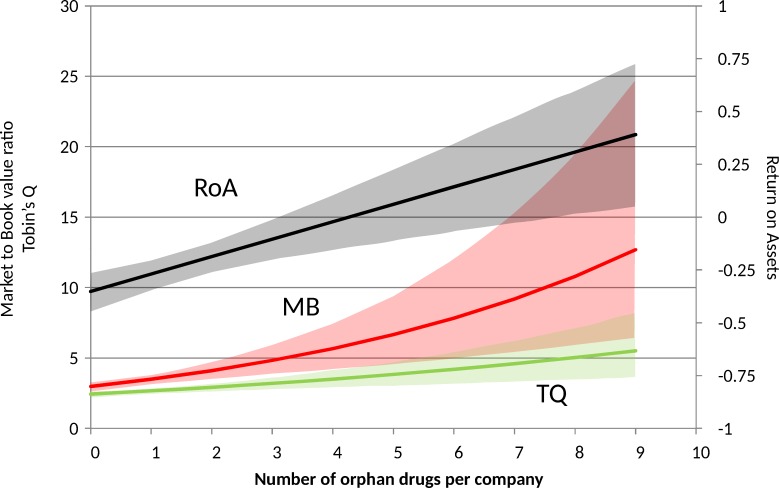
Relation between the number of orphan drugs marketed and the performance of companies. Calculated from predictions of the fitted pooled OLS regression, with NORPHAN as the explanatory variable of interest, and with all other covariates fixed to average values. Data are presented as the means and 95% confidence bounds (S4 Table).

We found a positive and significant relationship between the number of orphan drugs being sold (NORPHAN) and company performance (Table 5; Fig 1). This finding was consistent using both regression methods (S4 Table). Based on system GMM, for each additional orphan drug sold, TQ increased by 2.7% (95% CI, 0.2% to 5.2%), MB by 5.8% (95% CI, 0.7% to 10.9%) and ROA by 11.1% (95% CI, 0.6% to 21.3%). As with the primary analysis, orphan drug companies perform consistently and significantly better than non-orphan companies using DORPHAN as the key variable of interest (Table 5). Based on SORPHAN, we observed that for each additional 10% increase in orphan drug sales (as a proportion of total sales), TQ increased by 4.6% (95% CI, 1.2% to 7.9%), MB by 3.8% (95% CI, -0.4% to 7.9%) and ROA by 1.6 units (95% CI, 0.3 to 2.9) (Table 5).

**Table 5:** Sensitivity analyses using the number of orphans drugs sold per year (NORPHAN), a non-variant orphan dummy (DORPHAN), and the proportion of orphan drug sales to total sales (SORPHAN).

	NORPHAN			DORPHAN			SORPHAN		
Variables	ln(TQ)	ln(MB)	ROA	ln(TQ)	ln(MB)	ROA	ln(TQ)	ln(MB)	ROA
_ORPHAN	0.027 (0.036)	0.058 (0.026)	0.041 (0.037)	0.226 (0.011)	0.480 (0.002)	0.327 (0.027)	0.459 (0.008)	0.375 (0.075)	0.159 (0.018)
Size	-0.014 (0.227)	-0.002 (0.195)	0.070 (0.174)	-0.015 (0.004)	-0.021 (0.007)	-0.025 (0.044)	0.029 (0.503)	0.002 (0.718)	0.067 (0.038)
Leverage	0.139 (<0.001)	0.491 (0.196)	-0.562 (0.040)	0.175 (<0.001)	0.991 (<0.001)	-0.886 (<0.001)	0.158 (0.331)	-0.016 (0.984)	-0.123 (0.474)
R&D/TA	0.219 (0.158)	0.535 (0.080)	-0.294 (0.567)	0.383 (<0.001)	0.721 (<0.001)	-0.451 (<0.001)	1.061 (0.244)	-0.370 (0.606)	-0.954 (0.014)
Capex/PPE	-0.025 (0.820)	-0.141 (0.384)	0.530 (0.033)	0.188 (<0.001)	0.135 (0.016)	-0.148 (0.072)	-0.096 (0.798)	0.460 (0.537)	0.038 (0.826)
Dependent_t-1_	0.634 (<0.001)	0.615 (<0.001)	0.262 (0.041)				0.480 (<0.001)	0.582 (<0.001)	-0.005 (0.980)
Constant	0.427 (0.016)	0.486 (0.042)	-1.152 (0.158)	0.839 (<0.001)	0.868 (<0.001)	0.212 (0.198)	-0.234 (0.724)	0.120 (0.880)	-0.812 (0.084)
Observations	2,390	2,141	2,411	2,677	2,448	2,704	512	480	524
Number of cases: controls	84: 244	83: 229	83: 250	86: 258	84: 248	86: 258	27: 69	25: 63	27: 70
AR(2) test	(0.659)	0.893	(0.732)				(0.874)	(0.364)	(0.317)
Hansen test	(0.197)	(0.371)	(0.212)				(0.465)	(0.282)	(0.442)
Difference-in-Hansen	(0.535)	(0.212)	(0.904)				(0.426)	(0.147)	(0.917)
Number of instruments	263	263	288				49	49	94
Wald χ^2^ test				298.74 (<0.001)	113.26 (<0.001)	767.19 (<0.001)			

Values are estimators (unless specified otherwise) with P values in parentheses. All regressions include time dummies. _ORPHAN denotes NORPHAN, SORPHAN or DORPHAN for each dummy variable.

Refer to footnote of Table 4 for interpretation of the system GMM model validity tests (with NORPHAN and SORPHAN). The Taylor and Hausman model was applied for DORPHAN.

Instruments are set in NORPHAN from lags 3 to 7 for the ln(TQ) and ln(MB) models, and from lags 3 to 8 for the ROA model; and, in SORPHAN from lags 2 onwards (using the Stata *collapse* command) for the ln(TQ) and ln(MB) models; and from lags 3 to 4 for the ROA model.

## Discussion

Pharmaceutical companies with orphan drugs among their portfolio of products are more profitable and command a higher market value than companies not engaged in orphan drug development. Our analysis of publicly listed European and US companies which are orphan drug market authorisation holders, reveals that their market value (based on the Tobin’s Q ratio) and profitability is about 10% higher than matched non-orphan companies. These findings are robust to assumptions relating to the method of analysis and alternative specification of the variable representing orphan drug status.

Our findings contradict previous reports [11,25] which suggested that orphan drug companies are less profitable and less valuable than other pharmaceutical companies, and which the authors explain to be because of higher R&D expenses that decrease their net profit margin. Morel et al’s (2014) [11] analysis is based on comparisons of financial ratios among cohorts of only 6–7 companies, but is prone to bias for not controlling for the size of orphan drug companies, and is compromised by having control companies being themselves producers of orphan drugs.

The strengths of our analysis included the use of a standard performance model which allowed the inference of consistent and unbiased parameters based on the whole set of observations of publicly listed companies. To eliminate selection bias that could arise from financial performance, we included active, dead and suspended companies over the period of interest. The econometric analysis was robust to account for the endogeneity problem common to corporate finance models.

However, we recognise limitations relating to data in that some companies were omitted because of a lack of available financial information, and orphan drug sales data were available for less than a third of cases. We further limited our sample to publicly listed European and US companies. Orphan drugs legislations in other jurisdictions also make provisions for a range of incentives for development, and many developers of orphan drugs are private companies for which no financial data are available.

The higher value and profitability of orphan drug companies can be plausibly explained by several factors. Firstly are the incentives on offer to develop orphan drugs, of which market exclusivity is most important. This is for 10 years in Europe (plus an extra 2 years if paediatric development included) and 7 years (plus 6 months with paediatric indications) for each orphan designation in the USA. Incentives also include protocol assistance, reduced or waived regulatory fees and, in the USA, tax credits equal to 50% of clinical investigation expenses, and clinical trial subsidies [26].

Secondly, the cost of orphan drug research and development is lower than for non-orphan drugs. Phase III clinical trials of orphan drugs, for instance, are less expensive than non-orphan drug trials, at an average of US$95m versus US$219m per product approved [4]. Many orphan drugs (38%) are repurposed from other indications [27], which reduces uncertainty concerning their safety, accelerates time to market authorisation [6,28], and increases regulatory success rates [29,30]. Following authorization, the cost of marketing is comparatively small because the target populations of physicians and patients are themselves so small. This contrasts with marketing costs for medicines generally, which exceed R&D costs [31].

Thirdly, there are legitimate concerns about the use of orphan drugs for unapproved non-orphan indications [32]. This practice increases companies’ revenues, and may be seen as exploiting the incentives and the spirit of the Orphan Drug Act.

Fourthly is the pricing of orphan drugs. The mean price of the top 100 orphan drugs by sales in 2014 was more than 6 times higher, at $137,782 per patient per year, than of a comparable sample of non-orphans ($20,875) [4]. Despite the smaller rare disease market, the revenue generating potential of orphans is similar to non-orphan drugs [6]. Neither public nor private health plans have much leverage in negotiating the prices for orphan drugs. In the USA, nearly all orphan drugs are covered by private health insurance and social insurance programs such as Medicare, which provides cover for many adults with debilitating rare conditions [33]. Across Europe, orphan drugs often bypass health technology appraisal, or at least are subject to more lenient processes [34].

In conclusion, our analysis shows that policies directed towards incentivising orphan drug development have worked to the extent that companies are profiting excessively. This may have the adverse unintended consequence of directing R&D resources away from other areas of unmet clinical need, a situation which will likely be exacerbated as common diseases are increasingly reconsidered as multiple rare conditions, each eligible for orphan designation.

We recommend that EU and US orphan drug legislations should make provisions for incentives to be proportionate to the monetary success associated with marketing orphan drugs. Continuation of the status quo will make orphan drugs less affordable and companies more profitable.

## Supporting Information

S1 Table Mean values of financial variables for orphan companies, and non-orphan drug companies matched by maximum propensity score. (DOCX)

S2 Table Impact of orphan drug company status on company performance as estimated by EBITDA/REV. (DOCX)

S3 Table Sensitivity analysis using pooled OLS regression. (DOCX)

S4 Table Sensitivity analyses using pooled OLS for the number of orphans drugs sold per year (NORPHAN) and the proportion of orphan drug sales to total sales (SORPHAN). (DOCX)
