# Supplementary material for: Profitability and Market Value of Orphan Drug Companies: A Retrospective, Propensity-Matched Case-Control Study
Source: PLoS One. 2016 Oct 21;11(10):e0164681. doi: 10.1371/journal.pone.0164681 (PMC5074462; doi:10.1371/journal.pone.0164681)
Supplement: S1 Table — (DOCX) [file pone.0164681.s001.docx]

**S1 Table.** Mean values of financial variables for orphan companies, and non-orphan drug companies matched by maximum propensity score

| **Variable** | **Orphan companies** | **Non-orphan companies matched by maximum propensity score** | |
| --- | --- | --- | --- |
|  | Mean | Mean (P value) | Bias (%) |
| *Performance measure* |  |  | |
| TQ | 3.73 | 9.33 (<0.001) |  |
| MB | 4.94 | 4.08 (0.270) |  |
| ROA | -0.15 | -0.68 (<0.001) |  |
| *Explanatory variables* |  |  | |
| Size | 12.02 | 11.03 (<0.001) | 33.5 |
| Leverage | 0.32 | 0.43 (0.091) | -8.2 |
| R&D/TA | 0.20 | 0.26 (0.001) | -16.8 |
| Capex/PPE | 0.33 | 0.32 (0.527) | 2.8 |
| Pharmaceutical sub-sector | 0.57 | 0.63 (0.009) | -11.1 |
|  |  |  |  |
| Mean bias (%) |  |  | 14.5 |
| Median bias (%) |  |  | 11.1 |

P values indicate the significance of the difference in means between controls and the sample of orphan drug companies. The standardised percentage bias is shown before and after matching. This is the difference of the means between the case and control samples, expressed as a percentage of the square root of the average of the sample variances in the case and control groups.
