# Supplementary material for: Profitability and Market Value of Orphan Drug Companies: A Retrospective, Propensity-Matched Case-Control Study
Source: PLoS One. 2016 Oct 21;11(10):e0164681. doi: 10.1371/journal.pone.0164681 (PMC5074462; doi:10.1371/journal.pone.0164681)
Supplement: S2 Table — (DOCX) [file pone.0164681.s002.docx]

**S2 Table.** Impact of orphan drug company status on company performance as estimated by EBITDA/REV

|  | **Pooled OLS** | | **System GMM** | |
| --- | --- | --- | --- | --- |
| **Variables** | **Estimator** | **P value** | **Estimator** | **P value** |
|  |  |  |  |  |
| ORPHAN | 9.196 | 0.002 | 5.732 | 0.042 |
| Size | 0.031 | 0.951 | 0.370 | 0.730 |
| Leverage | -5.095 | 0.038 | -4.553 | 0.370 |
| R&D/TA | -51.241 | 0.003 | -84.906 | 0.030 |
| Capex/PPE | -12.711 | 0.031 | 3.351 | 0.766 |
| Constant | 4.610 | 0.484 | -1.112 | 0.950 |
| Dependent_t-1_ |  |  | 0.272 | <0.001 |
|  |  |  |  |  |
| Number of observations | 2,574 |  | 2,259 |  |
| Number of cases : controls | 85 : 255 |  | 80 : 238 |  |
| R-squared | 0.048 |  |  |  |
| Number of instruments |  |  | 283 |  |
| AR(2) test |  |  | 0.146 |  |
| Hansen test |  |  | 0.390 |  |
| Difference-in-Hansen test |  |  | 0.996 |  |

The pooled OLS regression includes time and country dummy variables, and t-statistics are based on robust, firm-clustered standard errors.

The system GMM regression includes time and country dummies. Instruments are set from t-4 onwards for the financial variables.

The number of companies included in the analysis was lower than the original sample of 86 cases and 258 controls. This was because system GMM requires at least two years of data, which were not available for some companies.

The AR(2) test reports the P value of a test for second-order serial correlation in the first-differenced residuals, under the null of no serial correlation. The Hansen test reports the P value of the test of over-identification under the null that all instruments are valid. The difference-in-Hansen test reports the P value of the null hypothesis that instruments used for the equations in levels are exogenous.
