# Supplementary material for: Profitability and Market Value of Orphan Drug Companies: A Retrospective, Propensity-Matched Case-Control Study
Source: PLoS One. 2016 Oct 21;11(10):e0164681. doi: 10.1371/journal.pone.0164681 (PMC5074462; doi:10.1371/journal.pone.0164681)
Supplement: S3 Table — (DOCX) [file pone.0164681.s003.docx]

**S3 Table.** Sensitivity analysis using pooled OLS regression

|  | **ln(TQ)** | **ln(MB)** | **ROA** |
| --- | --- | --- | --- |
| **Variables** | Estimator (P value) | Estimator (P value) | Estimator (P value) |
|  |  |  |  |
| ORPHAN | 0.241 (<0.001) | 0.425 (<0.001) | 0.280 (<0.001) |
| Size | -0.004 (0.366) | -0.003 (0.817) | -0.003 (0.851) |
| Leverage | 0.250 (<0.001) | 0.252 (0.209) | -1.076 (<0.001) |
| R&D/TA | 0.570 (<0.001) | 1.447 (<0.001) | -0.563 (0.060) |
| Capex/PPE | 0.308 (<0.001) | 0.343 (<0.001) | -0.143 (0.374) |
| Constant | 1.076 (<0.001) | 1.110 (<0.001) | -0.094 (0.618) |
|  |  |  |  |
| Number of observations | 2,677 | 2,448 | 2,704 |
| Number of cases : controls | 86 : 258 | 84 : 248 | 85 : 258 |
| R-squared | 0.278 | 0.154 | 0.380 |

All t-statistics are based on robust, firm-clustered standard errors. All regressions include time and country dummy variables.
