# Supplementary material for: Profitability and Market Value of Orphan Drug Companies: A Retrospective, Propensity-Matched Case-Control Study
Source: PLoS One. 2016 Oct 21;11(10):e0164681. doi: 10.1371/journal.pone.0164681 (PMC5074462; doi:10.1371/journal.pone.0164681)
Supplement: S4 Table — (DOCX) [file pone.0164681.s004.docx]

**S4 Table.** Sensitivity analyses using pooled OLS for the number of orphans drugs sold per year (NORPHAN) and the proportion of orphan drug sales to total sales (SORPHAN)

|  | **NORPHAN** | | | **SORPHAN** | | |
| --- | --- | --- | --- | --- | --- | --- |
| **Variables** | **ln(TQ)** | **ln(MB)** | **ROA** | **ln(TQ)** | **ln(MB)** | **ROA** |
|  |  |  |  |  |  |  |
| _ORPHAN | 0.091 (<0.001) | 0.162 (<0.001) | 0.083 (<0.001) | 0.847 (<0.001) | 1.050 (<0.001) | 0.238 (<0.001) |
| Size | -0.005 (0.571) | 0.003 (0.853) | -0.002 (0.913) | -0.009 (0.647) | -0.011 (0.693) | 0.052 (<0.001) |
| Leverage | 0.249 (<0.001) | 0.256 (0.212) | -1.077 (<0.001) | 0.234 (0.001) | 1.653 (0.001) | -0.175 (<0.001) |
| R&D/TA | 0.574 (<0.001) | 1.449 (<0.001) | -0.562 (0.060) | 0.903 (0.004) | 2.151 (0.002) | -1.339 (<0.001) |
| Capex/PPE | 0.313 (<0.001) | 0.354 (<0.001) | -0.141 (0.384) | 0.291 (0.038) | 0.394 (0.049) | 0.037 (0.639) |
| Constant | 1.092 (<0.001) | 1.138 (<0.001) | -0.084 (0.660) | 0.428 (0.178) | 0.126 (0.794) | -0.727 (0.003) |
|  |  |  |  |  |  |  |
| Observations | 2,677 | 2,448 | 2,704 | 587 | 554 | 600 |
| R-squared | 0.277 | 0.155 | 0.379 | 0.310 | 0.255 | 0.301 |
| Number of cases : controls | 86 : 258 | 84 : 248 | 86 : 258 | 28 : 68 | 28 : 68 | 28 : 74 |

Values are estimators (unless specified otherwise) with P values in parentheses. _ORPHAN denotes NORPHAN or SORPHAN for either dummy variable. All regressions include time and country dummies. T-statistics are based on robust, firm-clustered standard errors.
